# Roux-en-Y Gastric Bypass Versus Single Anastomosis Duodeno–Ileal Bypass With Sleeve Gastrectomy: Different Mechanisms, Similar Outcomes

**DOI:** 10.1155/jobe/4870532

**Published:** 2025-11-28

**Authors:** Hye Ju Shin, Chan Woo Kang, Eun Kyung Wang, Ye Bin Kim, Jung Ho Nam, Doyeon Kim, Yang Jong Lee, Ju Hun Oh, Cheol Ryong Ku

**Affiliations:** ^1^Department of Clinical Drug Discovery & Development, Yonsei University College of Medicine, Seoul, Republic of Korea; ^2^Department of Internal Medicine, Endocrinology, Institute of Endocrine Research, Yonsei University College of Medicine, Seoul, Republic of Korea; ^3^Brain Korea 21 PLUS Project for Medical Science, College of Medicine, Yonsei University, Seoul, Republic of Korea

**Keywords:** diabetes mellitus, glucotonic effect, Roux-en-Y gastric bypass, single anastomosis duodeno–ileal bypass with sleeve gastrectomy

## Abstract

**Objective:**

We aimed to compare the molecular mechanisms and metabolic outcomes of Roux-en-Y gastric bypass (RYGB) and single anastomosis duodeno–ileal bypass with sleeve gastrectomy (SADI-S) using a preclinical model.

**Methods:**

Otsuka Long-Evans Tokushima Fatty rats with diet-induced obesity underwent RYGB, SADI-S, or sham surgery. Metabolic parameters, including glucose tolerance, body weight, and 18F-fluorodeoxyglucose biodistribution, were assessed at 1- and 2-month postsurgery. The expression of Glucose transporter 1 (GLUT1) and glucose metabolism–related genes in intestinal segments was analyzed.

**Results:**

Although RYGB and SADI-S yielded comparable improvements in glucose tolerance and body weight at 1 month postsurgery, they exerted their effects through distinct mechanisms. RYGB enhanced GLUT1-mediated glucose excretion in the common limb, whereas SADI-S upregulated the expression of the glycolytic genes *Hk2, Fbp2, Aldob,* and *Ldha* in the colon. Two months postsurgery, the observed metabolic improvements diminished despite sustained weight loss, which coincided with decreased expression of GLUT1 and glycolytic genes.

**Conclusions:**

RYGB and SADI-S achieve similar benefits through distinct glucose handling pathways; however, these effects decline over time. Our data do not support the superiority of SADI-S over RYGB, particularly given its higher complication rate, and instead highlight the need for strategies aimed at prolonging the therapeutic benefits of metabolic surgeries.

## 1. Introduction

The fast-increasing global prevalence of metabolic disorders, notably obesity and Type 2 diabetes mellitus (T2DM), presents major public health and economic challenges [1, 2]. For individuals with severe obesity, lifestyle interventions and pharmacological approaches are often insufficient for long-term weight reduction and glycemic control. In such cases, metabolic surgery has emerged as the most effective intervention, providing sustained weight loss and improvements in metabolic parameters, including T2DM remission [3–5].

**Figure 1:**
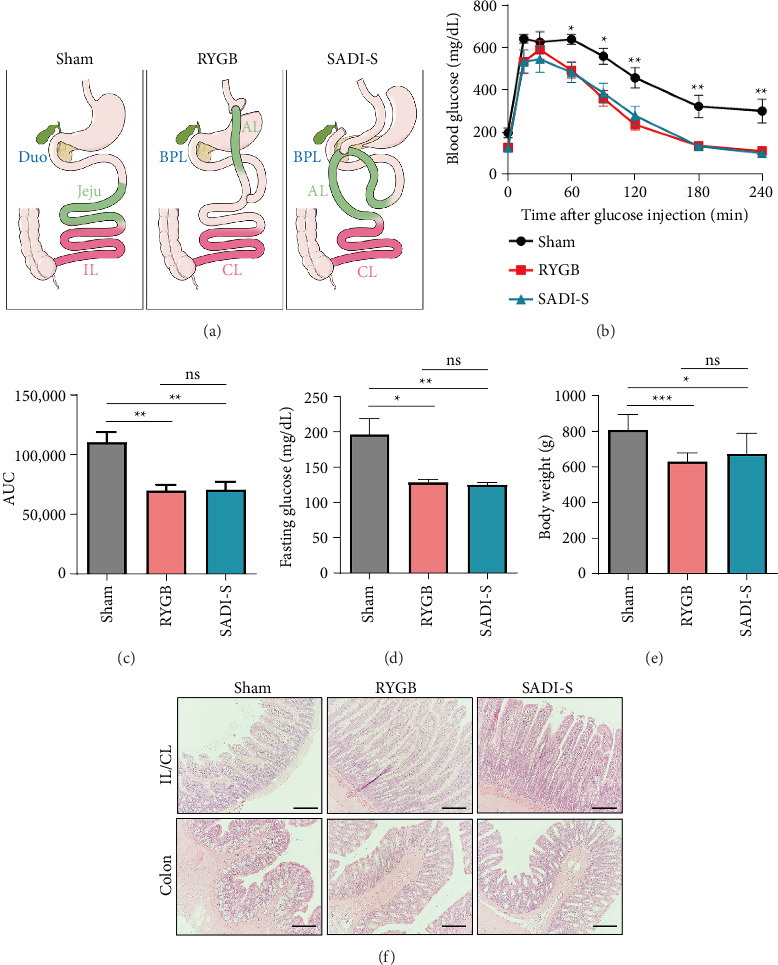
Roux-en-Y gastric bypass (RYGB) and single anastomosis duodeno–ileal bypass with sleeve gastrectomy (SADI-S) demonstrate similar outcomes in terms of improvements in metabolic parameters and changes in intestinal morphology. (a) Schematic diagram of sham, RYGB, and SADI-S procedures. The RYGB procedure was performed as we previously described [20]. The SADI-S procedure was performed as previously described [22]. Otsuka Long-Evans Tokushima Fatty (OLETF) rats were fed a high-fat diet (HFD) for induction of diet-induced obesity (DIO), underwent surgical procedures, and were monitored for 4 or 8 weeks postoperatively. The green segments indicate surgically rearranged jejunum (Jeju) intestinal segments, while the red segment represents the ileum (IL) in all groups. (b) Intraperitoneal glucose tolerance test (IPGTT) data after 1 month postsurgery in OLETF. RYGB (*n* = 11) and SADI-S (*n* = 12) group rats exhibit significantly improved glucose tolerance compared to sham (*n* = 11) group rats. (c) Area under the curve (AUC) analysis of the IPGTT results confirms reduced glucose excursions in both surgical groups, indicating enhanced glycemic responses. (d) Fasting blood glucose concentrations in RYGB, SADI-S, and sham groups. (e) Body weights in the RYGB and SADI-S groups are 20%–25% lower than those in the sham group. (f) Hematoxylin and eosin (H&E) staining demonstrates increased villus length and crypt depth in the ileum (IL) and colon of RYGB and SADI-S groups compared to those in the sham group. Scale bars of images indicate 100 μm. All data are presented as the mean ± SEM. Data in b, c, d, and e were analyzed using the Mann–Whitney *U* test; ^∗^*p* < 0.05, ^∗∗^*p* < 0.01, and ^∗∗∗^*p* < 0.001.

Among the various bariatric procedures, Roux-en-Y gastric bypass (RYGB) and sleeve gastrectomy are the most widely performed and extensively studied, with well-documented efficacy and safety profiles [6–9]. Recently, a single anastomosis duodeno–ileal bypass with sleeve gastrectomy (SADI-S) was developed to combine the benefits of duodenal switch and sleeve gastrectomy while reducing surgical complexity [10, 11]. SADI-S involves a single duodeno–ileal anastomosis and preserves pyloric function, which may improve nutrient absorption and reduce the risk of complications compared to traditional biliopancreatic diversion with duodenal switch [12] (Figure 1(a)).

Comparative clinical studies suggest that short-term outcomes of SADI-S, particularly the weight loss and T2DM remission, may surpass those of RYGB [5, 13, 14]. The technical simplicity and shorter operative time of SADI-S further enhance its appeal [10, 12]. However, SADI-S is also associated with a higher incidence of early postoperative complications, including sepsis and complications requiring reoperation, particularly during the surgical learning curve [11]. Furthermore, long-term outcome data for SADI-S remain scarce compared to those available for RYGB, which, despite being more established, has been associated with late-stage complications including internal hernia and marginal ulceration [13, 15].

Although clinical differences between RYGB and SADI-S are becoming increasingly clear, the molecular mechanisms underlying their metabolic effects remain poorly understood. Notably, recent studies have identified a key role of Glucose transporter 1 (GLUT1) in mediating intestinal glucose uptake following RYGB, contributing to glycemic control through insulin-independent pathways [16, 17]. Our previous research further established the “glucotonic effect,” the intestine's ability to absorb glucose from the circulation and excrete it into the lumen, as a novel mechanism underpinning the metabolic effects of RYGB [18–21]. These findings underscore the importance of intestinal glucose reprogramming, particularly alterations in GLUT1 activity in the small intestine, in driving metabolic improvements following bariatric surgery.

To date, no studies have directly compared RYGB and SADI-S in terms of the molecular reprogramming of intestinal glucose handling, which represents a major gap in our understanding of the mechanisms by which these surgeries exert their effects. Whether SADI-S alters GLUT1 activity in a similar way to RYGB, or employs distinct intestinal adaptations, remains unknown.

In this study, we sought to compare the metabolic effects of RYGB and SADI-S using a preclinical model of obesity. By integrating metabolic profiling, histological evaluation, and molecular analyses, we aimed to delineate the mechanistic divergence between these two procedures, with a specific focus on GLUT1-mediated glucose handling. Furthermore, we investigated the metabolic benefits of RYGB and SADI-S at 1- and 2-month postsurgery to address critical gaps in the mechanistic understanding of SADI-S and to identify potential therapeutic targets to sustain and enhance the effects of bariatric interventions.

## 2. Materials and Methods

### 2.1. Ethical Considerations

All animal procedures were approved by the Institutional Animal Care and Use Committee of the Severance Hospital, Seoul, Republic of Korea (IACUC approval no. 2022-0024) and performed in accordance with the relevant guidelines.

### 2.2. Establishment of the Animal Model of Obesity

Obesity was induced in male Otsuka Long-Evans Tokushima Fatty (OLETF) rats by providing a high-fat diet (HFD), comprising 60% fats, 20% carbohydrates, and 20% protein (D12492; Research Diets, Inc., New Brunswick, NJ, USA) starting from 5 weeks of age for 3 months. Following the induction of obesity, the rats underwent either a RYGB, SADI-S, or sham surgery.

### 2.3. Surgical and Postsurgical Procedures

Surgery was performed after an overnight fasting. During surgery, anesthesia was maintained by inhalation of 1%–4% isoflurane. Postoperatively, all animals received 20 mL/kg saline and 1 mg/kg meloxicam subcutaneously. Animals were fasted for 24 h postsurgery. After evaluating the health and behavior of each animal, a liquid diet was reintroduced 48–72 h postsurgery. A soft diet was provided after 2 days, and the rats resumed their preoperative diet on Postoperative day 7. Early surgical mortality (< 1 week postoperatively) was ∼60%, being almost entirely due to leakage of the gallbladder (GB)-to-bowel anastomosis regardless of its location. Surgical success rate, defined as rat survival for > 1 week without surgical complications, was 65% for RYGB and 55% for SADI-S. Surgical complications included obstruction at the site of the GB anastomosis to the jejunum or ileum (IL) (< 5%) and occurred within 1–4 weeks postsurgery. Taking these success rates into account, we assigned 12 animals per group, with 5-6 animals allocated for each of the 1- and 2-month postoperative timepoints. The chosen sample sizes reflect practical considerations based on surgical feasibility and anticipated survival, and we now acknowledge this limitation explicitly in the manuscript.

### 2.4. RYGB

The RYGB procedure was performed as we previously described [20]. A 5 cm midline incision was made to expose and identify the Treitz ligament. The entire length of the small intestine was measured from the Treitz ligament to the cecum. The jejunum was transected 20 cm distal to the Treitz ligament, creating the biliopancreatic limb (BPL) and alimentary limb (AL). The esophagogastric junction was carefully isolated from surrounding tissues, including gastric vessels and the vagus nerve, and the stomach was transected approximately 3 mm below this junction. An end-to-side gastrojejunostomy was performed to connect the gastric pouch to the AL using interrupted 6-0 polyglycolic acid sutures. The BPL was anastomosed to the small intestine 20 cm distal to the gastrojejunostomy, maintaining a BPL: AL: common limb (CL) ratio of 1:1:2.5. The laparotomy was closed in two layers using 4-0 silk sutures.

### 2.5. SADI-S

The SADI-S procedure was performed as previously described [22]. Following a midline incision, the stomach was separated from the pancreas and spleen, and approximately 70%–80% of it was clamped and resected; the remaining portion was closed using continuous 4/0 Monosyn sutures. The small intestine length was measured, and the duodenum was transected immediately distal to the bile duct entrance. A 5-mm opening was created in the remaining duodenal wall. The IL was identified at the halfway point of the small intestine, and a corresponding 5-mm hole was created. The duodenal and IL holes were then connected, creating a single anastomosis bypass route while preserving the full length of the common channel. The abdomen was closed in layers with 4-0 silk and 6-0 black sutures.

### 2.6. Intraperitoneal Glucose Tolerance Test (IPGTT)

Following an overnight fast, rats were weighed and injected intraperitoneally with a glucose solution (2-g glucose/kg body mass). Blood glucose concentrations were measured at 0, 15, 30, 60, 90, 120, and 240 min after glucose administration using a handheld glucometer (Arkray, Inc., Kyoto, Japan) by making an incision in the tail of each rat and gently massaging 35–50 μL blood onto a glucose test strip. Pre-op IPGTT was performed 1 week before surgery, and post-op IPGTT was conducted 1 week prior to sacrifice at 1- and 2-month postsurgery.

### 2.7. In Vivo 18F-Fluorodeoxyglucose (FDG) Excretion

At 1 month after surgery, IPGTT was performed after an overnight fast. Rats were sedated to reopen the abdominal cavity. The anastomosis sites of each reconstructed limb were ligated as follows: at the AL, BPL, and CL in RYGB group rats; at the sleeve-preserving duodenal stump and IL limb in SADI-S group rats; and at the IL and duodenum–jejunum segments in the sham group rats. This was performed to quantify the amount of radiotracer excreted into each individual limb and to prevent upstream radiotracer from entering the lumen of downstream segments. Immediately after ligation, the abdominal wall was sutured, and a radiotracer mixture (1 mCi 18F-FDG in 0.2-mL phosphate-buffered saline [PBS]) was injected into the tail vein. The rats were sacrificed 1 h after radiotracer injection, and the intestines were harvested en bloc.

For RYGB group rats, the intact alimentary tract was excised after identifying the suture lines at the gastrojejunostomy and end-to-side jejuno-jejunostomy sites. Small incisions were made in the BPL, AL, and CL, and approximately 40 mL of PBS was flushed through each limb, which were then harvested separately. For SADI-S group rats, similar incisions were made in the sleeve-preserving duodenal segment and IL limb, with 40 mL of PBS flushed through each segment and the common channel. For the sham group rats, the same total volume of PBS (120 mL) was flushed through the small intestine.

Gamma counting was performed separately for each limb PBS to determine the excreted FDG, with results reported as total radioactivity (counts per minute). Autoradiography images of the flushed intestines were acquired using a barium sulfide film (exposure time: 10 min) to determine the FDG distribution in each limb.

### 2.8. Histology and Immunohistochemistry

Tissues collected immediately after euthanasia were snap-frozen in liquid nitrogen and stored at −80°C or immersed in formalin at ambient temperature for 2 days. Formalin-fixed tissues were sent to the Animal Histology Core (Yonsei Bio-Medical Research Center, Seoul, Republic of Korea) for histology. Tissues were embedded in paraffin, and 4 µm-thick sections were used for hematoxylin and eosin (H&E) staining or immunohistochemistry to detect GLUT1. For immunohistochemistry, sections were dewaxed and rehydrated, and antigen retrieval was achieved by boiling in citrate buffer (pH 6). The sections were then incubated with a rabbit anti-GLUT1 primary antibody (colon 1:1500, CL/IL 1:500; ab115730, Abcam, Cambridge, UK). Subsequently, the sections were incubated with a secondary antibody polymer for 10 min (Leica Biosystems, Nussloch, Germany), developed with 3,3′-diaminobenzidine for 10 min, and counterstained with hematoxylin for 10 min. Images were acquired using a BX43 microscope (Olympus Corp., Tokyo, Japan).

### 2.9. Quantitative PCR

Total RNA was isolated from tissues using an RNeasy Mini purification kit (74104; QIAGEN, Hilden, Germany) according to the manufacturer's protocol. Tissues were homogenized in RLT buffer using a bead homogenizer (TissueLyser II; Qiagen) for 3 min at maximum speed. cDNA was prepared using ReverTra Ace (Toyobo, Osaka, Japan) and subjected to quantitative real-time PCR using Power SYBR Green PCR Master Mix (Applied Biosystems, Waltham, MA, USA) according to the manufacturers' instructions. The primers used are listed in Table S1.

### 2.10. Western Blot Analysis

Membrane and cytoplasmic protein fractions of tissue were obtained using a Mem-PER Plus Membrane Protein Extraction Kit (Thermo Fisher Scientific, Waltham, MA, USA). Lysates were supplemented with Protease Inhibitor Cocktail (P8340; Merck Group, Darmstadt, Germany), Phosphatase Inhibitor Cocktail 2 (P5726; Merck Group), and Phosphatase Inhibitor Cocktail 3 (P0044; Merck Group) and centrifuged at 16,000 *g* for 15 min at 4°C. Protein concentrations were quantified using a BCA Protein Assay Kit (71285-M; Merck Group). Lysates containing equal amounts of protein were mixed with NuPAGE LDS Sample Buffer (4X) (NP0007; Thermo Fisher Scientific) and boiled for 10 min at 70°C before being subjected to 4%–20% gradient SDS-PAGE using NuPAGE 4%–12% Bis-Tris Protein Gels (NP0336BOX; Thermo Fisher Scientific). Proteins were transferred to polyvinylidene fluoride membranes and probed with antibodies against GLUT1 (1:1000; ab115730; Abcam) and Na^+^/K^+^-ATPase (1:50000; ab76020; Abcam).

### 2.11. Statistical Analysis

All data are presented as mean ± standard error of the mean (SEM). For comparisons between two groups, the Mann–Whitney *U* test was applied, as it does not assume normality and is appropriate for small sample sizes. Statistical analyses were conducted using GraphPad Prism software Version 6 (GraphPad Software, La Jolla, CA, USA), and a *p* value < 0.05 was considered statistically significant.

## 3. Results

### 3.1. RYGB and SADI-S Result in Similar Short-Term Metabolic Outcomes and Intestinal Remodeling

1 month postsurgery, metabolic parameters significantly improved in both the RYGB and SADI-S groups compared to the sham group (Figure 1). IPGTT results demonstrated enhanced glucose tolerance in both the RYGB and SADI-S groups, with significant reductions in blood glucose levels observed 60 min in RYGB and SADI-S rats following glucose administration (Figure 1(b)). The area under the curve (AUC) analysis confirmed diminished glucose excursions in RYGB and SADI-S group rats (Figure 1(c)), alongside lower fasting blood glucose levels (Figure 1(d)); no significant intergroup differences were observed. Weight loss was comparable between the RYGB and SADI-S groups (Figure 1(e)), with body weights 20%–25% less than those observed in the sham group (*p* < 0.01), underscoring the comparable efficacy of the two surgical techniques in terms of short-term weight loss.

Histological analysis revealed adaptive intestinal remodeling in both surgical groups. H&E staining indicated increased villus length and crypt depth in the IL/CL and colon in rats from both surgical groups compared to sham-operated controls (Figure 1(f)); these differences were confirmed by quantitative morphometric data (Figure S1). These findings suggest that, despite differing anatomical configurations, RYGB and SADI-S induce analogous intestinal adaptations that may contribute to their metabolic benefits.

### 3.2. RYGB and SADI-S Differ in the Anatomical Localization of GLUT1-Mediated Glucose Homeostasis

**Figure 2:**
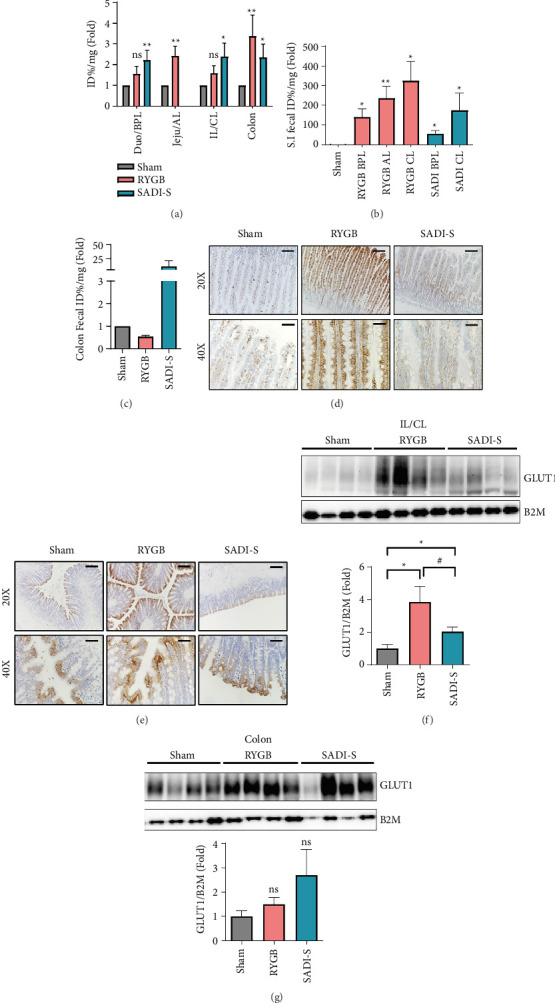
RYGB and SADI-S differ in the anatomical localization of GLUT1-mediated glucose homeostasis. (a) 18F-Fluorodeoxyglucose (FDG) biodistribution analysis shows the quantitative analysis of FDG uptake in the postwashing intestine tissue. Elevated glucose uptake across intestinal segments in both surgical groups compared with that in the sham group. Duodenum (Duo) vs. biliopancreatic limb (BPL), jejunum (Jeju) vs. alimentary limb (AL), ileum (IL) vs. common limb (CL). (b) Intestine phosphate-buffered saline (PBS) washing analysis. Small intestinal luminal FDG excretion is significantly increased in RYGB rats, particularly in the common limb. (c) Colon phosphate–buffered saline (PBS) washing analysis. SADI-S rats exhibit a pronounced increase in colonic luminal FDG excretion compared to the sham and RYGB groups. (d-e) Immunohistochemical (IHC) staining of GLUT1 in the small intestine (ileum or CL) and colon following each surgical procedure. (d) CL demonstrates robust GLUT1 upregulation in the RYGB group, with predominant basolateral localization. (e) IHC of the colon demonstrates increased GLUT1 expression in the SADI-S group, with broader apical distribution compared to that of the sham or RYGB groups. (f-g) Immunoblot analyses of the IL and colon, with GLUT1 expression levels normalized to beta-2 microglobulin (B2M). (f) GLUT1 expression was increased in both surgeries; however, the increase was relatively lower in the SADI-S group compared to the RYGB group. (g) In the colon, GLUT1 expression showed no statistically significant difference among the groups. The bar graphs below represent the relative GLUT1 intensity (GLUT1/B2M). All data are presented as the mean ± SEM. *n* = 6 in each group (a–c) and *n* = 4 in each group (f and g). Data in a, b, c, f, and g were analyzed using the Mann–Whitney *U* test; ^∗^*p* < 0.05, ^∗∗^*p* < 0.01, and ^#^*p* < 0.05; ns, nonsignificant.

To delineate the possible mechanisms underlying the improvements in glycemic control, we assessed intestinal glucose uptake and excretion using FDG biodistribution analysis. The RYGB and SADI-S group rats exhibited elevated glucose uptake across intestinal segments compared with that of the sham group rats (Figure 2(a)). However, luminal glucose excretion patterns differed significantly between the RYGB and SADI-S groups. RYGB group rats displayed a marked increase in FDG excretion into the small intestinal lumen, particularly in the CL, indicative of enhanced GLUT1-mediated luminal glucose excretion (Figure 2(b)). In contrast, SADI-S group rats showed a pronounced increase in colonic luminal FDG excretion (*p* < 0.05 vs. sham; Figure 2(c)), suggesting a shift in glucose handling toward the distal gut. Our previous study identified GLUT1 as a crucial mediator of luminal glucose excretion following RYGB [20], with heightened GLUT1 expression in intestinal cells postsurgery facilitating the absorption of circulating glucose from the bloodstream and its subsequent excretion into the intestinal lumen, significantly contributing to improved glycemic control. To determine whether a similar mechanism underlies the effects of SADI-S, we next examined GLUT1 expression in intestinal tissues using immunohistochemistry and western blotting. The RYGB group exhibited GLUT1 upregulation in the CL compared to the sham group, with expression predominantly located basolaterally (Figures 2(d) and 2(f)), consistent with a luminal glucose excretion effect. In contrast, the SADI-S group demonstrated elevated GLUT1 expression primarily in the colon, with broader apical distribution than that observed in the sham or RYGB groups (Figures 2(e) and 2(g)), highlighting a distinct anatomical focus for glucose transport. These data indicate that, despite the similar metabolic outcomes of RYGB and SADI-S, RYGB enhances glucose excretion in the small intestine, whereas SADI-S preferentially augments glucose handling in the colon.

### 3.3. SADI-S Preferentially Activates Colonic Glucose Metabolism

**Figure 3:**
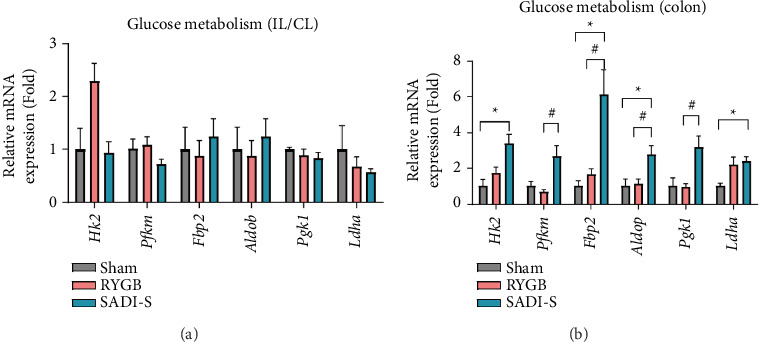
Roux-en-Y gastric bypass (RYGB) and single anastomosis duodeno–ileal bypass with sleeve gastrectomy (SADI-S) achieve metabolic benefits through different pathways: SADI-S upregulates colonic glucose metabolism pathway genes. (a) Quantitative real-time PCR (qRT-PCR) analysis of glucose metabolism–related genes in the IL and CL. Expression of glucose metabolism–related genes shows no significant difference between the sham and surgical groups. (b) qRT-PCR analysis of glucose metabolism–related genes in the colon. SADI-S rats exhibit significant upregulation of glycolytic genes in the colon, Hexokinase 2 (*Hk2*), Fructose-1,6-bisphosphatase 2 (*Fbp2*), aldolase B (*Aldob*), and lactate dehydrogenase A (*Ldha*), compared to both sham and RYGB groups. Expression levels were normalized to those of B2M. This coordinated upregulation indicates enhanced colonic glucose utilization and metabolic reprogramming specifically in the SADI- S procedure. All data are presented as the mean ± SEM. *n* = 5-6 in each group (a and b). Data in a and b were analyzed using the Mann–Whitney *U* test; ^∗^*p* < 0.05 and ^#^*p* < 0.05.

To further investigate the mechanistic divergence in glycemic control between RYGB and SADI-S, we performed a detailed analysis of intestinal glucose metabolism at the molecular level using quantitative real-time PCR. We first examined the expression of key glucose metabolism–related genes, including Hexokinase 2 (*Hk2*) and Fructose-1,6-bisphosphatase 2 (*Fbp2*), which are involved in glycolysis and gluconeogenesis, respectively. As shown in Figure 3(a), there were no significant differences in the expression of these genes in either the IL/CL among the sham, RYGB, and SADI-S groups. This suggests that neither surgical intervention alters local glucose metabolism in these regions.

In contrast, expression levels of *Hk2*, *Fbp2*, aldolase B (*Aldob*), and lactate dehydrogenase A (*Ldha*) were upregulated in SADI-S group colonic tissue compared to sham and RYGB group colonic tissue (*p* < 0.05 for all comparisons; Figure 3(b)). These genes collectively regulate glucose phosphorylation, glycolytic flux, and lactate production, indicating an overall increase in colonic glucose utilization in SADI-S group rats compared to sham and RYGB group rats. These metabolic genes were not upregulated in the colon of RYGB group rats compared to sham group rats, highlighting a fundamental mechanistic distinction between RYGB and SADI-S.

### 3.4. RYGB and SADI-S Result in Sustained Weight Loss but Divergent and Temporary Glucose Homeostasis

**Figure 4:**
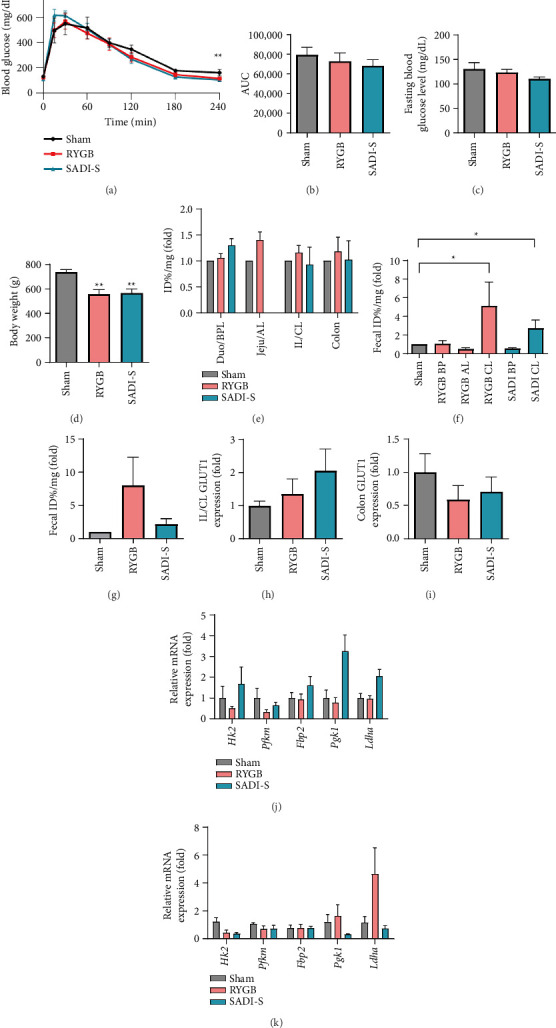
Progressive attenuation of the impact of bariatric surgery: Decreased efficacy in both Roux-en-Y gastric bypass (RYGB) and single anastomosis duodeno–ileal bypass with sleeve gastrectomy (SADI-S) groups at the 2-month follow-up. (a) Intraperitoneal glucose tolerance test (IPGTT) results at 2-month postsurgery show reduced glucose tolerance in both surgical groups compared to 1-month outcomes, with SADI-S showing improved tolerance only at the 240-min time point. (b) AUC analysis of IPGTT results. AUC values in both surgical groups are no longer significantly different from those of the sham group, indicating loss of metabolic benefits observed at 1 month. (c) Fasting blood glucose concentrations were measured using a hand-held glucometer after an overnight fast. Reduction in fasting glucose levels is attenuated, with only SADI-S maintaining significantly lower levels than those in the sham group. (d) Body weight measurements. Body weight loss remains significantly greater in both surgical groups than that in the sham group despite loss of metabolic benefits. (e) FDG biodistribution analysis shows the quantitative analysis of FDG uptake in the postwashing intestine tissue. Unlike the 1-month data, the surgery-induced increase in intestinal glucose uptake was no longer observed in either surgical group. (f-g) Quantification of luminal FDG excretion in (f) small intestinal and (g) colonic segments using PBS flush and gamma counting methods. Small intestinal and colonic luminal FDG excretion is decreased in both groups compared to measurements taken at 1 month, indicating loss of enhanced glucose excretion mechanisms. (h-i) qRT-PCR analysis of GLUT1 mRNA expression in the (h) small intestine (ileum and CL) and (i) colon. Expression levels were normalized to those of B2M. (j-k) qRT-PCR analysis of glucose metabolism–related genes (*Hk2, Pfkm, Fbp2, Pgk1,* and *Ldha*) in (j) small intestine and (k) colon. Expression of colonic glucose metabolism genes (*Hk2* and *Ldha*) in the SADI-S group is no longer significant from that in the sham group, with *Hk2* expression falling below the levels in the sham. Expression levels were normalized to that of B2M. All data are presented as the mean ± SEM. *n* = 5-6 in each group (a–k). Data in a and b were analyzed using the Mann–Whitney *U* test; ^∗^*p* < 0.05 and ^∗∗^*p* < 0.01.

To investigate the later-stage metabolic effects of bariatric surgery, glucose homeostasis and body weight were monitored for 2 months postsurgery. The metabolic improvements observed at 1 month were markedly diminished 2 months postsurgery. IPGTT results revealed impaired glucose tolerance in both the RYGB and SADI-S groups compared to their corresponding results at 1 month, except for improved tolerance at the 240-min time point in the SADI-S group (Figure 4(a)). Furthermore, the reductions in the AUC and fasting blood glucose levels previously observed in the RYGB group relative to the sham group were no longer evident (Figures 4(b) and 4(c)). Despite the attenuation of glucose homeostasis in the RYGB and SADI-S groups 2 months postsurgery, weight loss remained significantly greater in these groups than in the sham group (Figure 4(d)).

FDG biodistribution analysis 2 months postsurgery revealed reduced intestinal glucose uptake (Figure 4(e) and Figure S3) and excretion (Figures 4(f) and 4(g)) in the RYGB and SADI-S groups than those observed 1 month postsurgery (Figures 2(a), 2(b)).

At the molecular level, GLUT1 expression in the CL of RYGB group rats decreased to near-baseline levels (Figure 4(h)), with colonic GLUT1 expression even lower than that in sham group rats (Figure 4(i)). In the SADI-S group, the expression levels of glucose metabolism–related genes in the colon were no longer significantly different from those of the sham group, with *Hk2* mRNA expression falling below that observed in the sham group (Figures 4(j) and 4(k)).

Collectively, these findings suggest that although RYGB and SADI-S initially induce distinct glucose handling mechanisms that improve metabolic parameters, these effects are significantly attenuated, although not completely abolished, over time. Despite sustained weight loss, the reduction in the expression of GLUT1 and glucose metabolism–related genes highlights a temporal limitation in the metabolic efficacy of these surgical interventions.

## 4. Discussion

This study provides a detailed comparative analysis of the metabolic effects and underlying mechanisms of RYGB and SADI-S in a preclinical HFD-induced obesity model. Our findings highlight the similarities and distinctions between these two bariatric procedures, providing new insights into the tissue-specific regulation of intestinal glucose metabolism and the temporal dynamics that influence later-stage metabolic outcomes.

At 1 month postsurgery, rats in both the RYGB and SADI-S groups exhibited significantly improved glucose tolerance, fasting blood glucose levels, and body weight compared to those of sham controls, consistent with the results of previous clinical and preclinical studies [23]. These findings underscore the short-term effectiveness of both surgical procedures. However, a deeper examination of glucose handling mechanisms revealed anatomical and molecular differences between the two procedures.

RYGB was associated with enhanced GLUT1 expression, primarily in the CL of the small intestine, accompanied by increased luminal excretion of glucose. These findings support the previously described “glucotonic effect,” in which systemic glucose is taken up by enterocytes and excreted into the intestinal lumen via basolaterally localized GLUT1, contributing to glycemic control independent of insulin [18–20, 24]. In contrast, rather than relying primarily on glucose excretion, SADI-S induced the significant upregulation of glycolytic enzymes, *Hk2, Fbp2, Aldob*, and *Ldha*, in the colon and elevated the expression of apically localized GLUT1 in colonic epithelial cells. This pattern suggests that local glucose utilization and luminal glucose clearance occur in parallel and identifies the colon as a metabolically active site contributing to glycemic regulation after SADI-S. GLUT1-mediated intestinal glucose reprogramming is not a phenomenon exclusive to bariatric surgery. Previous studies have reported that metformin administration in mice and patients also improves metabolism through serum blood glucose excretion into the gut lumen in both the small and large intestines [24–26]. These findings suggest that intestinal glucose metabolism reprogramming has significant implications for metabolic improvement.

The differences between the RYGB and SADI-S groups are likely due to their distinct surgical configurations. RYGB preserves a longer AL and separates food from biliopancreatic secretions earlier in the jejunum, whereas SADI-S retains a longer common channel and bypasses more of the small intestine, directing more unabsorbed nutrients toward the colon. As a result, metabolic reprogramming following RYGB occurs primarily in the small intestine, whereas SADI-S shifts the metabolic focus distally to the colon.

Following both RYGB and SADI-S, initial metabolic improvements were markedly reversed 2 months postoperatively and coincided with diminished GLUT1 expression and decreased glycolytic gene expression in the small intestine of RYGB group rats and the colon of SADI-S group rats. This suggests that the observed reprogramming of intestinal glucose metabolism is temporary. Notably, clinical studies have also documented the recurrence of T2DM after initial remission following bariatric surgery, indicating that the loss of metabolic benefits over time is not limited to animal models [27–29]. Although different in species and experimental context, these findings collectively support the idea that surgical effects on glucose regulation may diminish with time. Our data further suggest a clear link between reduced glucose homeostasis observed during the 2-month IPGTT and the concomitant decrease in GLUT1 expression and glycolytic gene activity. Interestingly, body weight reduction was maintained throughout the study period, implying that the reprogramming of intestinal glucose metabolism may play a more central role in glycemic improvement than in weight loss.

The waning efficacy observed could be attributed to several physiological compensatory mechanisms, such as adaptive changes in nonresected intestinal segments, decreased secretion of hormones such as glucagon-like peptide-1, or epithelial remodeling that reverses initial changes in gene expression [30–32]. The colonic upregulation of glucose metabolism–related genes in the SADI-S group was particularly transient, suggesting that the distal gut may be more susceptible to these compensatory mechanisms. These findings call into question the durability of the metabolic benefits of SADI-S, especially as reports have suggested that the rates of early postoperative complications are higher following SADI-S than following RYGB [14, 33].

Taken together, our results emphasize that although both RYGB and SADI-S exhibit comparable short-term benefits via distinct glucose handling mechanisms, neither surgical procedure achieves sustained molecular reprogramming of intestinal glucose metabolism. Thus, the notion that SADI-S induces metabolic effects superior to those of RYGB cannot be supported, particularly in light of its potential for later-stage adverse events. Rather than supporting the use of one procedure over the other, our findings highlight the importance of understanding temporal changes in postsurgical physiology. These findings suggest that GLUT1 overexpression in the intestinal tract following surgery may contribute to the rapid improvement in hyperglycemia observed after metabolic procedures. Intestinal glucose metabolism reprogramming potentially offers novel insights for the development of antidiabetic and weight loss medications.

Our study has some limitations. First, the relatively short follow-up period limits our ability to fully characterize the long-term metabolic consequences of RYGB and SADI-S. While we observed a significant attenuation of metabolic benefits 2 months after surgery, longer-term preclinical and clinical data would help determine whether this trend continues or stabilizes. Second, our study was conducted in a rat model of HFD-induced obesity, which may not fully reflect the complex pathophysiology of human metabolic disease. Third, while we identified GLUT1-mediated glucose handling as a key mechanism, we did not explore potential interventions to sustain or enhance this process. Future studies should investigate whether upregulation of intestinal GLUT1 expression through pharmacological or genetic approaches can prolong the metabolic benefits observed following bariatric surgery, emphasizing GLUT1 as a shared and actionable metabolic target across distinct intestinal regions. Integrating microbiome analyses and epithelial lineage tracing [34–37] may offer strategies to extend the therapeutic window. Furthermore, combining metabolic surgery with pharmacological agents, such as glucagon-like peptide-1 receptor agonists, may help reinforce the initial benefits and counteract physiological adaptations [38, 39].

## 5. Conclusions

Our study demonstrates that RYGB and SADI-S improve glucose metabolism through anatomically distinct GLUT1-mediated mechanisms in the intestine; however, these beneficial effects are significantly attenuated over time. Given the lack of a sustained advantage and the higher procedural burden associated with SADI-S, our data do not support its superiority over RYGB. These findings underline the need for long-term mechanistic studies to refine surgical interventions and inform precision strategies to treat metabolic diseases.

## Data Availability

The data that support the findings of this study are available from the corresponding author upon reasonable request.

## Supporting Information

Supporting Figure S1. Quantitative analysis of villus length and crypt depth in the IL and CL after RYGB and SADI-S surgery compared to sham controls.

Supporting Figure S2. Differential GLUT1 expression and regional FDG uptake in the intestine 1 month after RYGB and SADI-S surgery.

Supporting Figure S3. Autoradiographic images showing normalized intestinal FDG uptake at 2 months postsurgery, with no significant differences among sham, RYGB, and SADI-S groups.

Supporting Table S1. Primer sequences used for quantitative PCR analysis of glucose transporters and glycolytic/gluconeogenic enzymes in *Rattus norvegicus*.
