# Supplementary material for: Roux-en-Y Gastric Bypass Versus Single Anastomosis Duodeno–Ileal Bypass With Sleeve Gastrectomy: Different Mechanisms, Similar Outcomes
Source: J Obes. 2025 Nov 28;2025:4870532. doi: 10.1155/jobe/4870532 (PMC12680477; doi:10.1155/jobe/4870532)
Supplement: Supporting Information — Additional supporting information can be found online in the Supporting Information section. [file 4870532.f1.docx]

Supplementary Materials


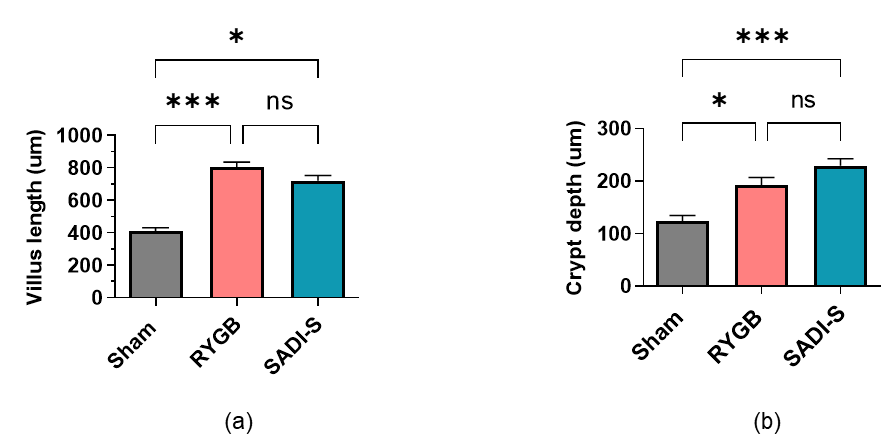


**Figure S1.** (a) Quantitative analysis of villus length in the ileum (IL) and common limb (CL) segments. Histological sections were stained with hematoxylin and eosin (H&E). Both RYGB and SADI-S groups exhibited significantly increased villus length compared to sham controls, indicating enhanced intestinal morphological adaptation following bariatric surgery.

(b) Quantitative analysis of crypt depth in IL and CL segments. Both surgical groups showed significantly increased crypt depth compared to sham controls, suggesting structural remodeling of the intestine that may contribute to improved metabolic outcomes. All data are presented as mean ± SEM, n=6 animals per group. Statistical analysis was performed using the Mann–Whitney U test for (a) and (b); **p* < 0.05, *** *p* < 0.001.

**
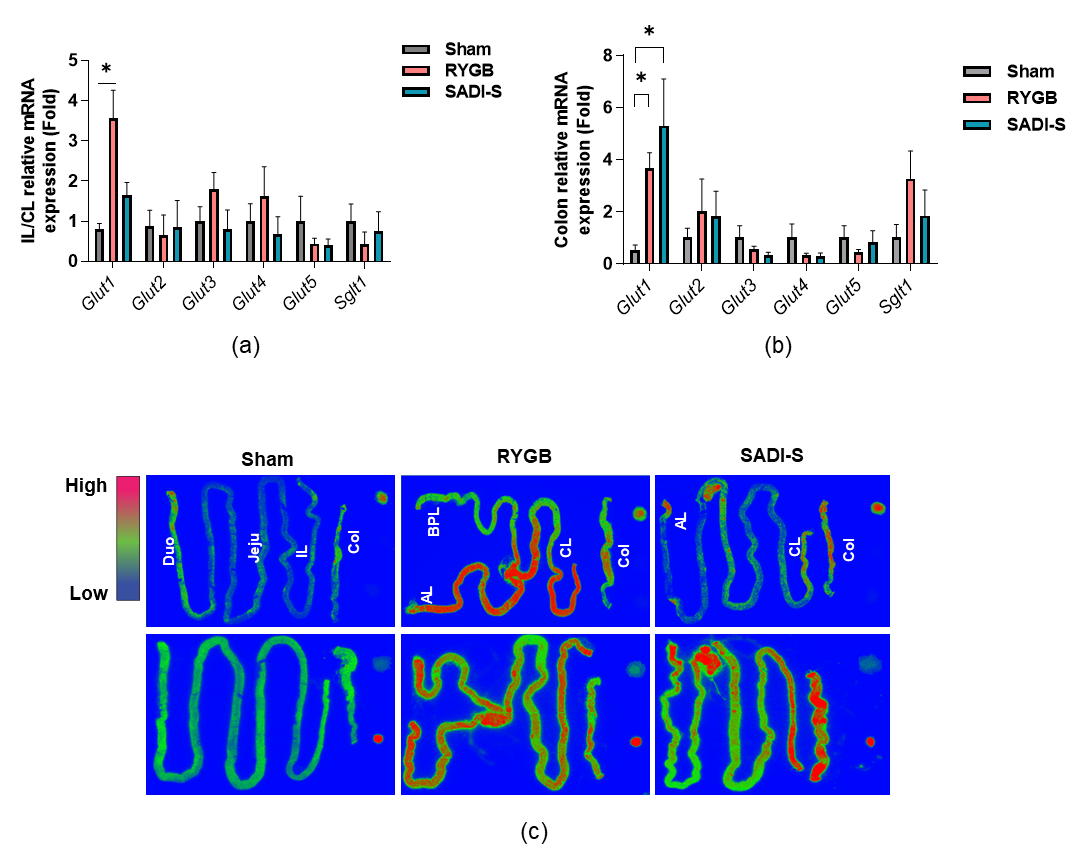
**

**Figure S2.** (a) Quantitative real-time PCR (qRT-PCR) analysis of glucose transporter mRNA in the ileum (IL) and common limb (CL). GLUT1 expression was significantly higher in the RYGB group than in sham and SADI-S. No significant differences were found in GLUT2–5 and SGLT1 among groups. (b) qRT-PCR analysis in the colon. GLUT1 was increased in both surgical groups vs. sham. The SADI-S group showed a non-significant trend toward higher expression than RYGB. No significant differences were observed in GLUT2–5 and SGLT1.
(c) Representative autoradiographic images of FDG uptake in the intestine one-month post-surgery. Red indicates high uptake; green indicates low. RYGB shows increased uptake in the small intestine (CL), while SADI-S shows higher uptake in the colon. Images are representative of n = 6 animals per group. All data are presented as mean ± SEM. Statistical analysis was performed using the Mann–Whitney U test for (a) and (b); **p* < 0.05.


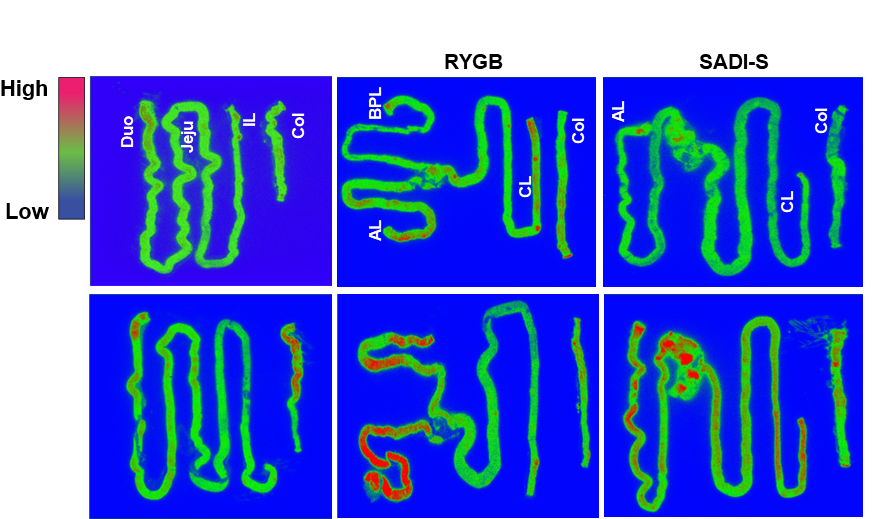


**Figure S3.** Representative autoradiographic images of FDG uptake in the small intestine and colon at two-month post-surgery. Red indicates relatively high FDG uptake; green indicates relatively low uptake. Compared to one-month post-surgery (Figure S2c), FDG accumulation in the RYGB group’s CL and in the SADI-S group’s colon was no longer prominently increased. No marked differences in FDG uptake were observed among sham, RYGB, and SADI-S groups at this time point. Images are representative of n = 5-6 animals per group.

**Table S1**

| **Gene** | **Species** | **Forward sequence (5’→3’)** | **Reverse sequence (5’→3’)** |
| --- | --- | --- | --- |
| ***B2m*** | Rattus norvegicus | CCAGTCCTTGCTGAAGGACAG | CCTCAACTGCTACGTGTCTCAG |
| ***Glut1*** | Rattus norvegicus | GTGCTCGGATCCCTGCAGTTCG | GGGATGGACTCTCCATAGCGTG |
| ***Glut2*** | Rattus norvegicus | TTGCCCTGACTTCCTCTTCCAACT | TAGTCAGATTGCTGGCCTCAGCTT |
| ***Glut3*** | Rattus norvegicus | GCGCAGCCCTTCCGTTTTGC | CGCTGGAGGATCTCCGTCGC |
| ***Glut4*** | Rattus norvegicus | GGTTTCACCTCCTGCTCTAA | TGGCATGGGTTTCCAGTATG |
| ***Glut5*** | Rattus norvegicus | TCGCACTGGCACTGCAGAACA | GCCCCACGGCGTGTCCTATG |
| ***Sglt1*** | Rattus norvegicus | GCCTACGGAACTGGAAGCTG | GACGGTGACGACGCTGATAG |
| ***Sglt2*** | Rattus norvegicus | AACTCAAAAGCAGTATAAGG | ACATGCCCTGGTTGCAACTC |
| ***Hk2*** | Rattus norvegicus | TGATCGCCTGCTTATTCACGG | AACCGCCTAGAAATCTCCAGA |
| ***Pfkm*** | Rattus norvegicus | GGCGGAGGAGAGCTAAAACT | CCCTGACCGCAGCATTCATA |
| ***Fbp2*** | Rattus norvegicus | GCTGTCCAATTCCCTGGTGA | TCCATCCAGTGGGTCAAAGC |
| ***Aldob*** | Rattus norvegicus | CCCTTCCAGCCTTGCTATCC | GGCAGTGCTCTAGGTCATGG |
| ***Pgk1*** | Rattus norvegicus | TTCCGCATCTCCATTTGGTGT | TCCCGATGCAGTAAAGACGA |
| ***Ldha*** | Rattus norvegicus | GCAGTTGGCAGTGTGTCTTG | GAGCTGTGGTTGGTCCAGTT |
